# An Indigenous food sovereignty initiative is positively associated with well-being and cultural connectedness in a survey of Syilx Okanagan adults in British Columbia, Canada

**DOI:** 10.1186/s12889-021-11229-2

**Published:** 2021-07-16

**Authors:** Rosanne Blanchet, Malek Batal, Louise Johnson-Down, Suzanne Johnson, Okanagan Nation Salmon Reintroduction Initiatives, Colette Louie, Eliza Terbasket, Pauline Terbasket, Howie Wright, Noreen Willows

**Affiliations:** 1 Department of Agricultural, Food & Nutritional Science, Faculty of Agricultural, Life & Environmental Sciences, University of Alberta, Edmonton Clinic Health Academy, 11405 87 Ave, Mailbox #54, Edmonton, AB T6G 2P5 Canada; 2 Département de nutrition, Faculté de Médecine, Université de Montréal, Pavillon Liliane de Stewart, CP 6128 succ. Centre-Ville, Montréal, QC H3T 1A8 Canada; 3 Centre de recherche en santé publique de l’Université de Montréal et du CIUSS du Centre-Sud-de-l’Île-de-Montréal [CReSP], 7101 Avenue du Parc, Montréal, QC H3N 1X7 Canada; 4 Okanagan Nation Alliance, 3535 Old Okanagan Hwy, West Kelowna, BC V4T 3L7 Canada; 5 Osoyoos Indian Band, 1155 SenPokChin Blvd, Oliver, BC V0H 1T8 Canada; 6 Lower Similkameen Indian Band, 1420 BC-3, BC V0X 1C3 Cawston, Canada

**Keywords:** Cultural food security, Food sovereignty, Traditional food, Cultural connectedness, Salmon, Indigenous, Well-being

## Abstract

**Background:**

For the Syilx Okanagan Nation in Canada, salmon has vital nutritional, cultural, and spiritual significance. Yet, the Okanagan Sockeye salmon population came to near extinction, resulting in a drastic decline in salmon consumption from high historical levels. Thus, restoring and protecting salmon is crucial to Syilx well-being and way of life. A Syilx-led food sovereignty initiative re-established the Okanagan Sockeye salmon population, which has resulted in a rise in fish harvesting. The aim of this study was to assess whether engaging with this initiative was associated with health, well-being, and cultural connectedness (i.e., degree to which one is integrated in their culture) among Syilx adults. Eating Okanagan Sockeye salmon was conceptualized as a proxy for engaging with this Indigenous food sovereignty initiative.

**Methods:**

265 Syilx adults completed a survey including a traditional food frequency questionnaire and questions on health status (e.g., BMI, self-assessed physical health), well-being (e.g., life satisfaction, stress levels), and cultural connectedness (e.g., sense of belonging, importance of cultural practices). Participants were divided into 3 groups based on their wild salmon eating during the year prior to the survey: (1) adults who ate Okanagan Sockeye salmon, (2) adults who ate salmon but did not usually know the species of the salmon they ate, or who solely ate salmon that were not Okanagan Sockeye; and (3) adults who did not eat any salmon.

**Results:**

A statistically significant gradient was observed for enhanced well-being and cultural connectedness, with individuals in group 1 having better indicators than those in group 2, and adults in groups 1 and 2 having better indicators than adults in group 3. No differences were observed in physical health outcomes between the three groups.

**Conclusion:**

Findings suggest that the initiative to re-establish Okanagan Sockeye salmon in the Okanagan River system may have led to better well-being and cultural connectedness among Syilx adults. This study highlights the importance of Indigenous food sovereignty as a way to enhance well-being and cultural connectedness among First Nations in Canada. Findings also reinforce the importance of assessing health and well-being in a wholistic way in Indigenous health research.

## Background

Since time immemorial, traditional food (TF; e.g., fish, fowl, game, and plants harvested from local Indigenous food systems) has been the primary source of food of First Nations, an Indigenous people recognized by the government of Canada. Nowadays, an important proportion of First Nations’ diet has been replaced by highly processed store-bought foods [1–3]. This nutrition transition was largely a forced act of survival by First Nations communities in reaction to multiple factors such as colonization, dispossession of traditional land, restricted access to fisheries, and ecosystem destruction, all of which limited First Nations’ food sovereignty [4–6]. Food sovereignty is considered the right of peoples to define their own food systems which are ecologically, socially and culturally appropriate [7]. The systemic persecution endured by First Nations and its negative consequences on availability and access to TF resulted in a disconnection from Indigenous cultural practices, poor nutrition, and a disproportionally high level of food insecurity, obesity, nutrition-related chronic diseases, and ill-being [5, 8–14]. The systemic racism and oppressive colonial policies and practices that led to the decrease in TF-related activities still exist and continue to adversely affect First Nations’ communities, territories and watersheds. This manuscript will provide an overview of the importance of TF, cultural practices, cultural connectedness and Indigenous food sovereignty to First Nations. It will then describe a Syilx-led Indigenous food sovereignty initiative and assess whether engaging with this initiative was associated with health, well-being, and cultural connectedness among Syilx adults. Lastly, it will discuss the importance of findings for First Nations.

Our research team consists of Indigenous members of the Syilx Nation and non-Indigenous academics who advocate for increased Indigenous food sovereignty in Canada and worldwide.

First Nations benefit from TF in numerous ways beyond its nutritional value. TF provides important cultural benefits through its harvesting, processing, distribution, preparation, and consumption [6, 8, 10, 15]. For instance, harvesting TF provides physical activity and strengthens connections to traditional territories and the natural world [16, 17]. Similarly, TF consumption contributes to (re)building bonds between First Nations individuals through food sharing practices and feasts [10, 18]. Indigenous cultural practices are intrinsically linked with the natural world and TF systems [4, 8, 10, 17, 19, 20]. Fishing is considered an integral component of culture for many First Nations communities [5, 21] and culture is an important determinant of First Nations’ health [22]. Strong cultural identity, cultural connectedness and cultural continuity, three concepts related to engagement and integration with one’s culture, have been associated with First Nations’ health and well-being [23–29]. Cultural connectedness refers to the degree to which one feels integrated with their culture, as well as the strength of their cultural connections [26]. Cultural identity, spirituality, attendance of cultural events and participation in traditional activities are important aspects of cultural connectedness [26, 27].

Considering that many of the challenges experienced by First Nations are due to decreased cultural connectedness [23, 25–28], and that cultural connectedness and the health and well-being of First Nations are deeply intertwined with their food systems [4, 8, 10, 19, 20], it is critical for First Nations to reclaim their food sovereignty. Indeed, restoring, maintaining, and protecting Indigenous food systems is, for First Nations, foundational to preserving their culture and improving their health and well-being [6, 15, 17, 19–21, 30–32]. Many First Nations communities, including the Syilx, are doing so by asserting their self-determination, strengthening their relationships with the land and natural world, and by upholding their responsibilities towards their land and watersheds [5, 6, 19, 21, 33–36].

Indigenous food sovereignty describes the right of Indigenous peoples to practice ancient cultural activities such as hunting, fishing, trapping, plant harvesting, and agriculture in certain areas to sustain themselves [32]. It emphasizes the revitalization of Indigenous food systems, cultural practices and ecological knowledge systems [6, 17, 34]. It also recognizes that TF is fundamental to self-determination and is a sacred and central piece to the upholding of relationships with the land, each other and the natural world [5, 20, 32]. Indigenous food sovereignty can be seen as a pathway towards decolonizing land, water and people [34] as it “privilege[s] Indigenous worldviews, livelihoods, and governance” [19]. Therefore, Indigenous food sovereignty is foundational to improving Indigenous people’s food security, nutrition and well-being, and to preserving their governance systems and ways of knowing and living [15, 19–21, 30–32].

**Fig. 1:**
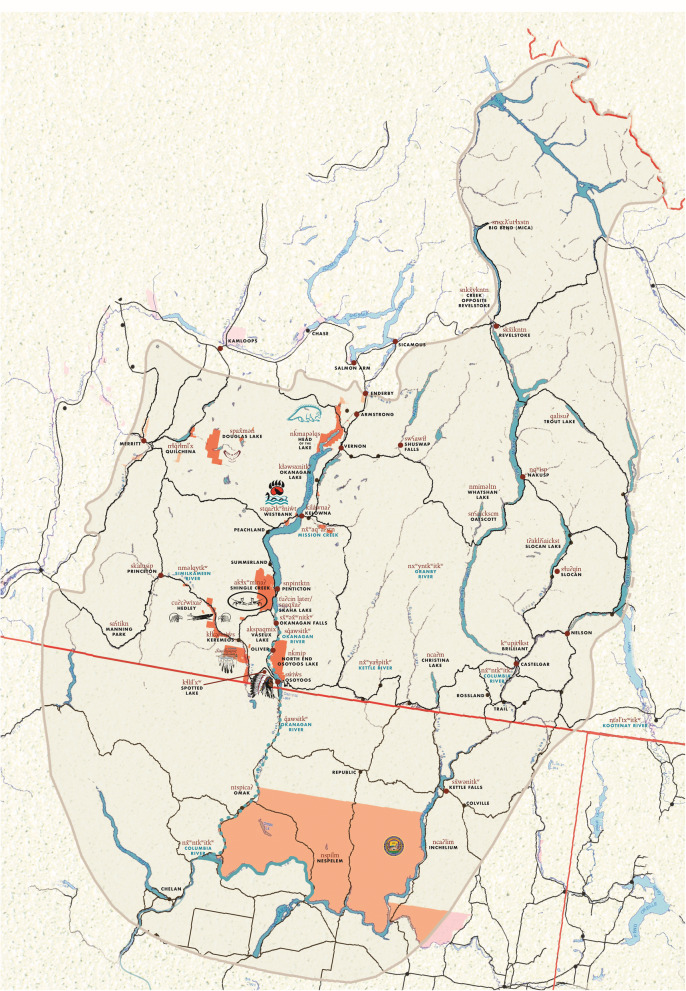
Okanagan Nation Alliance. (2017) View of the Syilx Okanagan Nation territory map. https://www.syilx.org/about-us/syilx-nation/territory/

Historically, the Syilx Okanagan First Nations (referred to as Syilx from now on) were subsistence fishers, hunters, and gatherers [37, 38]. Their traditional land and watersheds expanded beyond the current colonial confines of their reserves and the geopolitical boundary of Canada (Fig. 1). Their diet mostly comprised wild salmon, game, berries, and plants [37, 38]. Salmon, including Okanagan Sockeye salmon (*Oncorhynchus nerka*) which is a distinct salmon species that spawns in the Okanagan River Basin, is central to Syilx culture, nutrition, and trade traditions—its importance is reflected in its position as one of four Food “Chiefs” that is central to Syilx culture and trade traditions [38–40]. Thus, the presence of salmon on the Syilx traditional territory is necessary for the continuation of Syilx language and cultural teachings. However, overfishing, damming projects on the Columbia River, poor water management strategies, and environmental disruptions dramatically decreased the salmon populations in the Columbia River system and its Okanagan sub-basin, resulting in near salmon extirpation by the 1990s [38, 40, 41]. Without a well-functioning ecosystem and adequate fish habitat, the salmon fishery could not be sustained [40]. This severely impacted Syilx food security and sovereignty over their Indigenous food systems and contributed to the disruption of physical and social well-being of Syilx people. Under the direction of Syilx Elders, the Okanagan Nation Alliance (ONA), a First Nation government that represents Syilx communities in Canada and the US, worked with their member communities on the 12-year (2004–2016) *Sockeye Salmon Reintroduction to Skaha Lake Project* to reclaim their food sovereignty and to improve availability of salmon in the Okanagan River Basin [39]. This project rebuilt Okanagan Sockeye salmon populations through yearly fish fry releases, restoring natural habitats and migratory pathways, and controlling water levels [42]. This Syilx-led initiative also included cultural activities and practices, such as prayers, salmon ceremonies and salmon feasts [39]. Since 2012, there is evidence that fish harvesting has increased [41, 43] and ancillary projects have promoted access to salmon through cultural activities aimed at improving fishing techniques and promoting intake of the reintroduced salmon [37].

There are still relatively few reports that describe the impact of Indigenous food sovereignty interventions on health, well-being and cultural connectedness of Indigenous individuals. This is especially true for interventions on fisheries–an element of food sovereignty that has been generally overlooked by the food sovereignty scholarship [5, 21]. Therefore, the aim of this study was to assess whether engaging with the Syilx-led *Sockeye Salmon Reintroduction to Skaha Lake Project* was associated with enhanced health, well-being, and cultural connectedness among Syilx adults. Cultural connectedness was used to exemplify various concepts related to engagement and integration with Syilx culture. Eating Okanagan Sockeye salmon was conceptualized as a proxy for engaging with this Indigenous food sovereignty initiative. Because of the cultural centrality of salmon, eating it as a proxy for engaging with food sovereignty was intuitively established by the group based on Syilx knowledge of diverse individual experiences with salmon and eating salmon as a common factor.

## Methods

The *Salmon and Our Health Study* is a research project designed at the request of ONA to evaluate the *Sockeye Salmon Reintroduction to Skaha Lake Project*. It is a community-based research partnership between the ONA, Syilx communities, the University of Alberta, and Université de Montréal. The overall objective of this study was to document health and health equity outcomes of the reintroduction of Okanagan Sockeye salmon in the Okanagan River Basin, British Columbia, Canada. Although the Syilx Okanagan Nation territory straddles the Canada-United States border, only communities in Canada were included in this research.

All researchers of the present study practice decolonizing research that honours Indigenous worldviews and cultural values. As such, this action-oriented research used a decolonizing health promotion framework. The researchers critically reflected on how colonial policies and practices impact Syilx well-being; honoured, applied and preserved Syilx knowledge, beliefs and practices through active participation of Syilx knowledge owners, including Elders; acknowledged Syilx knowledge as a form of intellectual property requiring attribution, co-participation and remuneration for knowledge sharing; and ensured that the research took collective action to undo the detrimental effects of the colonial legacy on Syilx food sovereignty [44].

Four authors (RB, MB, LJD, NW) are non-Indigenous settler academics living in Canada who position themselves as community-based participatory researchers. Of the two Syilx authors, one (SJ) is a Syilx scholar and the other (ONSRI) represents the study advisory group which is constituted of Syilx/Okanagan Nation members and other Indigenous people working for the Okanagan Nation Alliance. Our collective aim was to co-create knowledge that could be used by the Syilx Nation and other Indigenous peoples to further enact their self-determination and reclaim their food sovereignty. Our ultimate goal was to contribute to reducing dietary and health inequities experienced by Indigenous people.

To ensure an effective and respectful research approach, this study followed the First Nations ethical Principles of Ownership, Control, Access and Possession (OCAP®) and the Canadian “Tri-Council Policy Statement: Ethical Conduct for Research Involving Humans” [45, 46]. Participating communities, the ONA, University of Alberta, and Université de Montréal signed community research agreements that ensured respectful and equitable power-sharing relations between parties. Participating communities and the ONA were involved in the planning of study activities and in the interpretation of research findings to ensure their relevance and cultural acceptability. Participating communities and the Nation own their data. Findings were shared with community members before being widely distributed. This study has been performed in accordance with the Declaration of Helsinki. All participants gave written informed consent. Ethics approval was obtained from the Research Ethics Office of the University of Alberta (Pro00067679) and the Research Ethics Committee of the Université de Montréal (16–074-CERES-D).

All seven Syilx communities located in Canada were invited to participate in the study; three agreed to do so. Data was collected between February and August 2018. Households were randomly selected in large communities (≥250 households) and all households were selected in smaller communities (< 250 households). Participants were required to be 19 years and older, to self-identify as Syilx or be in a kin relationship with a person who self-identified as Syilx, and to be living on a Syilx reserve or an adjacent town. Pregnant and breastfeeding women were excluded. When more than one household member was eligible, the one with the next birthday was selected. Community interviewers were instructed to contact all households assigned to them. In total, 561 households were selected, 329 (58%) were contacted, three households were ineligible, six homes were vacant resulting in 320 eligible households. Of those, 265 households completed the survey, representing a participation rate of 82.8%.

Community interviewers were trained and supported by (1st author, RB), who is a Registered Dietitian with experience conducting research with Indigenous communities. The survey was developed and pilot-tested with community members, it was further refined with community interviewers’ feedback. The survey collected information on dietary patterns, lifestyle, physical and mental health status, well-being, cultural practices, and the importance of salmon to participants. Wild salmon eating was assessed using a traditional food frequency questionnaire (TFFQ) that estimated intake for the year prior to the survey. The TFFQ asked participants to identify the type of the salmon i.e., any type, Okanagan Sockeye, non-Okanagan Sockeye, Okanagan Chinook, non-Okanagan Chinook, Chum, Pink or Coho for each season.

The 18-item USDA Household Food Security Survey Module adapted to Indigenous populations in Canada was used to assess household food security status (food secure: 0 affirmative responses; food insecure: 1or more affirmative responses) [47]. Cultural food security was assessed by asking participants if they worried that traditional food would run out before they could get more (often, sometimes, never) and how important traditional food was in ensuring their family had enough to eat (very important, somewhat important, not very important, not important) [48].

Indicators of physical health status included body mass index (BMI; calculated using measured or reported weight and height) and weight status (normal-weight, overweight, obesity; based on BMI cut-offs) [49]. There were no statistically significant differences between measured (*n* = 161) or reported (*n* = 55) BMI (data not shown). Participants reported their self-assessed physical health status (poor, fair, good, very good, excellent), and whether they had been diagnosed with diabetes, hypertension or cardiovascular disease (yes, no). They also reported on their physical activity (less active than average, average, above average), and whether they smoked the day prior to the survey (yes, no). Well-being was assessed with self-assessed mental health status (poor, fair, good, very good, excellent), stress level (extremely stressful, quite a bit stressful, a bit stressful, not very stressful, not at all stressful), and life satisfaction (very satisfied, satisfied, neither satisfied not dissatisfied, somewhat dissatisfied, very dissatisfied) as asked in the Canadian Community Health Survey (CCHS) [50].

Questions designed to be proxies of cultural connectedness were developed with community members using in-house questions and questions from CCHS [50] and the Regional Health Survey [51]. These included a sense of belonging to the community (very strong, somewhat strong, medium, somewhat weak, very weak), wanting more TF (yes, no), wanting to go on the land more often (yes, no), TF gathered by household members in the past year (yes, no), referring to TF in *nsyilxcn* (the Syilx language) (always, often, sometimes, rarely, never), used traditional cooking methods in the past year (always, often, sometimes, rarely, never), using traditional storage methods for TF (yes, no), individual cultural identity linked to salmon (strongly agree, agree, neutral, disagree), Okanagan salmon more important for them than other salmon (yes, no), household ever participated in salmon feast or fish fry release ceremonies (yes, no), awareness of ONA’s efforts to restore the Sockeye salmon population in the Okanagan River Basin (yes, no), proud of their Nation’s relation to salmon (strongly agree, agree, neutral, disagree), importance of cultural practices (e.g., the act of reciprocity, returning the salmon bones to the water, grief protocols; very important, important, somewhat important, not very important, not important), and used traditional medicine in the past year (yes, no).

To assess the engagement with this Indigenous food sovereignty initiative, participants were divided into 3 groups based on their wild salmon eating during the previous year as reported in the TFFQ: Adults who did not eat any salmon; adults who ate salmon but did not usually know the species of the salmon they ate or who ate solely salmon that were not Okanagan Sockeye; and adults who ate Okanagan Sockeye salmon. All statistical analyses were conducted using SAS 9.4 (SAS Institute Inc. Cary, NC, USA). Means are presented with a 95% confidence interval and were compared by bivariate regression analyses with the categories of salmon eating as the independent variable. Chi-square tests were used for proportions.

## Results

Mean age of participants was 49.8 years and 70% were women (Table 1). In the year prior to the survey, almost half (47.9%) of respondents ate Okanagan Sockeye salmon, with the remainder eating other salmon species (37.0%) or not eating salmon (15.1%). There were few differences in sociodemographic characteristics between the three groups. The ratio of men to women was higher among Okanagan salmon eaters, followed by other salmon eaters and by non-salmon eaters, respectively (*p* = 0.0041). Although there were no differences in overall household food security status among the groups, a statistically significant gradient was observed for both measures of cultural food security status. Okanagan Sockeye eaters were the least worried that TF would run out before they could get more, and the most likely to consider that TF was important in ensuring that their family had enough to eat, followed by other salmon eaters and non-salmon eaters.

**Table 1:** Sociodemographic characteristics by salmon eating in Syilx Okanagan adults in British Columbia, Canada

		Salmon eating^1^			
Characteristic	Total	None	Type not specified or solely not Okanagan Sockeye	Okanagan Sockeye	P^2^
N	265	40	98	127	
Gender (n (%))					0.0041
Women	186 (70.2)	33 (82.5)	76 (77.5)	77 (60.6)	
Men	79 (29.8)	7 (17.5)	22 (22.5)	50 (39.4)	
Age (years) (mean (95% CI))	49.8 (47.7–51.7)	46.4 (41.2–51.6)	49.1 (45.5–52.6)	51.2 (48.5–53.9)	0.7118
Household Size (mean (95% CI))	3.48 (3.24–3.72)	3.53 (2.90–4.15)	3.63 (3.22–4.04)	3.35 (3.02–3.69)	0.5483
Households with Children (< 18 years) (n (%))					0.5163
Yes	138 (52.5)	21 (55.3)	55 (56.1)	62 (48.8)	
No	125 (47.5)	17 (44.7)	43 (43.9)	65 (51.2)	
Households with older adults (> = 65 years) (n (%))					0.2951
Yes	68 (25.9)	6 (15.8)	28 (28.6)	34 (26.8)	
No	195 (74.1)	32 (84.2)	70 (71.4)	93 (73.2)	
Household main source of income (n (%))					0.8919
From salary/self-employment	164 (64.6)	24 (66.7)	63 (65.6)	77 (63.1)	
Other	90 (35.4)	12 (33.3)	33 (34.4)	45 (36.9)	
Working for salary (n (%))					0.9234
Yes	216 (87.5)	32 (80.0)	81 (82.5)	103 (81.1)	
No	49 (18.5)	8 (20.0)	17 (17.4)	24 (18.9)	
Schooling (n (%))					0.7171
Less than high school diploma or its equivalent	26 (10.2)	5 (13.9)	9 (9.18)	12 (9.84)	
High school diploma or its equivalent or higher	230 (89.8)	31 (86.1)	89 (90.8)	110 (90.2)	
Food security status (n (%))					0.4418
Food secure	140 (54.3)	17 (44.7)	54 (55.7)	69 (56.1)	
Food insecure	118 (45.7)	21 (55.3)	43 (44.3)	54 (43.9)	
Worried traditional food would run out (n (%))					0.0083
Never	94 (37.1)	19 (50.0)	39 (41.5)	36 (29.7)	
Sometimes	107 (42.3)	8 (21.1)	35 (37.2)	64 (52.9)	
Often	52 (20.6)	11 (28.9)	20 (21.3)	21 (17.4)	
Importance of traditional food in ensuring family has enough to eat (n (%))					< 0.0001
Yes (somewhat to very important)	207 (80.5)	19 (50.0)	75 (78.1)	113 (91.9)	
No (not, not very important)	50 (19.5)	19 (50.0)	21 (21.9)	10 (8.13)	

CI confidence interval. ^1^During the year prior to the survey. ^2^Bivariate regression analyses or chi-square test

Physical health indicators (weight status, self-assessed physical health, and presence or absence of chronic disease) were not associated with salmon eating, regardless of species or harvest location (Table 2). Among well-being indicators, a statistically significant gradient was observed for enhanced well-being with Okanagan Sockeye eaters having better indicators than other salmon eaters, and both groups of salmon eaters having better indicators than non-salmon eaters, specifically for stress levels and life satisfaction. A similar gradient was observed for cultural connectedness proxies across the three groups (Table 3). The gradient was statistically significant for sense of belonging to their community, wanting to go on the land, TF gathered by household members in the past year, individual cultural identity linked to salmon, importance of cultural practices, referring to TF in *nsyilxcn*, used traditional medicine in the past year, ever participated in an Okanagan salmon feast or fish fry release ceremony by a household member, being aware of the Nation’s efforts to increase Sockeye salmon and used traditional cooking methods in the year before the survey.

**Table 2:** Health and well-being characteristics by salmon eating in Syilx Okanagan adults in British Columbia, Canada

		Salmon eating^1^			
Characteristic	Total	None	Type not specified or solely not Okanagan Sockeye	Okanagan Sockeye	P^2^
N	265	40	98	127	
BMI (kg/m^2^) (mean (95% CI))	31.0 (30.1–31.9)	33.1 (29.7–36.5)	30.3 (28.9–31.8)	30.9 (29.8–32.1)	0.0874
Weight status (n (%))					0.4523
Normal-weight	87 (32.8)	17 (42.5)	34 (34.7)	36 (28.3)	
Overweight	59 (22.3)	6 (15.0)	23 (23.5)	30 (23.6)	
Obesity	119 (44.9)	17 (42.5)	41 (41.8)	61 (48.0)	
Physical health (n (%))					0.2913
Poor/fair	101 (39.2)	19 (50.0)	38 (39.2)	44 (35.8)	
Good/very good/excellent	157 (60.8)	19 (50.0)	59 (62.8)	79 (64.2)	
Diabetes (n (%))					0.9682
Yes	38 (14.8)	5 (13.5)	14 (14.7)	19 (15.2)	
No	219 (85.2)	32 (86.5)	81 (85.3)	106 (84.8)	
Hypertension (n (%))					0.5200
Yes	55 (21.5)	9 (24.3)	17 (17.7)	29 (23.6)	
No	201 (78.5)	28 (75.7)	79 (82.3)	94 (76.4)	
Cardiovascular disease (n (%))					0.4007
Yes	13 (5.1)	3 (8.1)	6 (6.3)	4 (3.3)	
No	243 (94.9)	34 (81.9)	90 (93.7)	119 (96.7)	
Physical activity (n (%))					0.1228
Less active than average	58 (22.7)	13 (35.1)	22 (22.7)	23 (19.0)	
Average or above	197 (77.3)	24 (64.9)	75 (77.3)	98 (81.0)	
Smoking (n (%))					0.9650
Yes	80 (30.8)	11 (29.0)	30 (30.9)	39 (31.2)	
No	180 (69.2)	27 (71.0)	67 (69.1)	86 (68.8)	
Mental health (n (%))					0.4378
Poor/fair	45 (17.5)	9 (23.7)	18 (18.6)	18 (14.9)	
Good/very good/excellent	211 (82.4)	29 (76.3)	79 (81.4)	103 (85.1)	
Stress (n (%))					0.0353
Stressful (extremely, quite a bit, a bit)	170 (67.2)	24 (64.9)	73 (76.8)	73 (60.3)	
Not very/ not at all stressful	83 (32.8)	13 (34.1)	22 (23.2)	48 (39.7)	
Life satisfaction (n (%))					0.0445
Yes (very, satisfied)	213 (83.5)	28 (75.7)	75 (78.9)	110 (89.4)	
No (neither, somewhat and very dissatisfied)	42 (16.5)	9 (24.3)	20 (21.0)	13 (10.6)	

CI confidence interval. ^1^During the year prior to the survey. ^2^Bivariate regression analyses or chi-square test

**Table 3:** Cultural connectedness indicators by salmon eating in Syilx Okanagan adults in British Columbia, Canada

		Salmon eating^1^			
Characteristic	Total	None	Type not specified or solely not Okanagan Sockeye	Okanagan Sockeye	P^2^
N	265	40	98	127	
Sense of belonging to community (n (%))					0.0026
Medium, somewhat, very strong	203 (79.3)	26 (68.4)	70 (72.2)	107 (88.4)	
Somewhat, very weak	56 (20.7)	12 (31.6)	27 (27.8)	14 (11.6)	
Wants more traditional food (n (%))					0.0071
Yes	229 (88.1)	29 (78.4)	82 (83.7)	118 (94.4)	
No	31 (11.9)	8 (21.6)	16 (16.3)	7 (5.60)	
Wants to go on the land more often (n (%))					0.0503
Yes	228 (89.1)	28 (80.0)	85 (86.7)	115 (93.5)	
No	28 (10.9)	7 (20.0)	12 (13.3)	8 (6.5)	
Traditional food gathered by household members (n (%))					0.0001
Yes	159 (60.0)	12 (30.0)	59 (60.2)	88 (69.3)	
No	106 (40.0)	28 (70.0)	39 (39.8)	39 (30.7)	
Refers to traditional foods in nsyilxcen^3^ (n (%))					0.0001
Always/often/sometimes	166 (64.3)	10 (27.0)	59 (60.8)	97 (78.2)	
Rarely/never	92 (35.7)	27 (73.0)	38 (39.2)	27 (21.8)	
Used traditional cooking methods (n (%))					< 0.0001
Always/often/sometimes	70 (27.1)	3 (8.33)	17 (17.3)	50 (40.3)	
Rarely/never	188 (72.9)	33 (91.7)	81 (82.7)	74 (39.4)	
Uses traditional storage for traditional food (n (%))					0.6259
Yes	21 (8.14)	4 (10.8)	6 (6.19)	11 (8.87)	
Not available	237 (91.9)	33 (89.2)	91 (93.8)	113 (91.1)	
Cultural identity linked to salmon (n (%))					0.0006
Agree (strongly, agree)	167 (64.5)	17 (45.9)	56 (57.1)	94 (75.8)	
Disagree/neutral	92 (35.5)	20 (54.0)	42 (42.9)	30 (24.2)	
Okanagan salmon more important than other salmon (n (%))					0.0891
Yes	177 (70.8)	25 (67.6)	60 (63.8)	92 (77.3)	
No	73 (29.2)	12 (32.4)	34 (36.2)	27 (22.7)	
Household ever participated in an Okanagan salmon feast or fish fry release ceremony (n (%))					0.0002
Yes	189 (73.0)	21 (56.8)	63 (64.3)	105 (84.7)	
No	70 (27.0)	16 (43.2)	35 (35.7)	19 (15.3)	
Awareness of efforts to increase Sockeye salmon (n (%))					0.0032
Yes	228 (88.0)	30 (81.1)	80 (81.6)	118 (95.2)	
No	31 (12.0)	7 (18.9)	18 (18.4)	6 (4.8)	
Proud of Nation’s relation to salmon (n (%))					0.2214
Agree (strongly, agree)	213 (82.9)	29 (78.4)	76 (79.2)	108 (87.1)	
Disagree/neutral	44 (17.1)	8 (21.6)	20 (20.8)	16 (12.9)	
Importance of cultural practices (n (%))					0.0004
Yes (somewhat to very important)	238 (93.3)	29 (78.4)	91 (94.8)	118 (96.7)	
No (not, not very important)	17 (6.7)	8 (21.6)	5 (5.2)	4 (3.3)	
Used traditional medicine in the past year (n (%))					0.0019
Yes	157 (59.2)	16 (40.0)	53 (54.1)	88 (69.3)	
No	108 (40.8)	24 (60.0)	45 (45.9)	39 (30.7)	

CI confidence interval. ^1^During the year prior to the survey. ^2^Chi-square test. ^3^Syilx Okanagan language

## Discussion

This study sought to assess whether engaging with the Syilx-led *Sockeye Salmon Reintroduction to Skaha Lake Project,* an Indigenous food sovereignty initiative, was associated with enhanced health, well-being, and cultural connectedness among Syilx adults. Findings showed that eating salmon was linked with well-being and cultural connectedness for Syilx adults, and that this association was strongest when Okanagan Sockeye salmon was eaten. On the other hand, there were no associations between salmon eating and physical health outcomes, regardless of species or harvest location. Given that eating Okanagan Sockeye salmon was conceptualized as a proxy for engaging with this Indigenous food sovereignty initiative, these findings suggest that Indigenous food sovereignty initiatives may enhance well-being and a sense of cultural connectedness more rapidly than physical health. It is also possible that individuals with enhanced well-being and cultural connectedness were more likely to engage with this Indigenous food sovereignty initiative. Several First Nations communities in Canada strive to reclaim their food sovereignty by restoring their TF systems [6, 19, 21, 33, 34, 36, 52]. Unfortunately, the impact of Indigenous food sovereignty initiatives on health, well-being and cultural connectedness of individuals and communities is still rarely assessed empirically, especially when the initiatives relate to fisheries [5, 21]. Therefore, the present study brings an important contribution to the literature on Indigenous food sovereignty.

The current study findings support that engaging in Indigenous food sovereignty initiatives contributes to (re)building communities [18] through enhanced cultural connectedness, an increased sense of belonging and improved well-being. By revitalizing Indigenous food systems, Indigenous food sovereignty initiatives aim to increase availability of and access to TF, and to contribute to the reinstatement of relationships between First Nations, their traditional land and watersheds, and the natural world [17, 19, 20, 32]. Most participants in the current study reported that their cultural identity was linked with salmon, with this association being stronger among Okanagan Sockeye eaters. This result is aligned with previous literature showing that TF have a central role in maintaining the cultural identity of First Nations [6, 8, 10, 15]. Results from the current study are also in agreement with findings from other research that documented associations between TF consumption or harvesting and cultural connectedness [8, 28, 29]. For instance, all Cree adults who participated in the study conducted by Godin Laberge et al. (2015), reported that the consumption of traditional Cree foods was an important part of the Cree identity and that eating traditional foods helped them connect with their culture [8]. Similarly, Anishinaabe adults mentioned that culturally meaningful activities such as hunting, fishing, and trapping, were factors supporting their cultural connectedness [29].

The lack of association between the consumption of Okanagan Sockeye salmon and physical health outcomes is similar to what has been reported in the literature. Indeed, TF consumption has often been associated with enhanced nutrition [2, 3, 53] but rarely with physical health outcomes [16, 54]. This lack of statistically significant association between TF eating and health measures may be explained by the health measures used, which may not be sufficiently sensitive, or because people with health concerns tend to replace less healthy store-bought with TF as a means to improve their health [8, 55]. It is also possible that Okanagan Sockeye salmon has not returned for long enough or in great enough abundance to lead to quantifiable differences in physical health outcomes. That is, the lack of association between salmon eating and physical health may be because an insufficient quantity of salmon is consumed or salmon is consumed infrequently. Therefore, it is possible that an impact will be measurable in the future. Still, findings from the present study reinforce the importance of including multiple indicators when assessing health of Indigenous Peoples to reflect a wholistic way of appraising health encompassing more than physical health. By using conventional health indicators, such as weight status and presence or absence of a chronic disease, Indigenous food sovereignty initiatives may be evaluated as not having health benefits. In other words, when emphasizing physical health indicators, one risks missing the impact of interventions on well-being and cultural connectedness, important aspects of the wholistic definition of health shared by Indigenous Peoples [23]. Cultural and well-being indicators may reveal a powerful story, as is the case in this study.

### Strengths and limitations

The current findings should be interpreted while taking the study strengths and limitations into account. This study was developed in partnership with Syilx community members from its inception, founding a meaningful community-based participatory research study. The proxies used were created by the team which comprises Syilx partners and were deemed by them to have face validity. Syilx partners reviewed findings and their interpretation to ensure their cultural relevance. The current study is also the first one to assess the impact of an Indigenous food sovereignty initiative on health, well-being and cultural connectedness among First Nations in Canada. On the other hand, causation or the direction of the influence could not be ascertained because of the cross-sectional nature of this study. The three communities that participated in the study may have been different from the four that did not participate. Also, only 58% of selected households were contacted by local community interviewers. This may have introduced a selection bias that may limit generalizability of the findings to all Syilx adults. Furthermore, the results may not be representative of the general male Syilx population because fewer men than women participated in the study [56]. Cultural connectedness is an abstract construct and the current study used proxy measures to assess it. Therefore, it is possible that proxies used might not be meaningful or applicable to all participants. Future work should aim to replicate the study findings using other meaningful ways to assess cultural connectedness. Similarly, we assessed the engagement with this Indigenous food sovereignty initiative using a proxy. Food choices are determined by several factors, such as food allergies and income, [10, 57] and may not necessarily represent the extent to which a person was engaged with the Sockeye salmon reintroduction initiative. In other words, there is a small number of Syilx who engage with this food sovereignty initiative but do not eat salmon. Lastly, salmon consumption depends on salmon availability and the salmon run in the Okanagan River Basin in 2017–2018 was low, which may have impacted the current findings.

## Conclusions

Findings from this study suggest that the initiative to bring Okanagan Sockeye back in the Okanagan River system may have led to better well-being and cultural connectedness among Syilx adults. It is also possible that individuals with enhanced well-being and cultural connectedness were more likely to engage with this Indigenous food sovereignty initiative. This study highlights both the importance of Indigenous food sovereignty and the cultural benefits of engaging with a culturally significant food for Syilx adults. It also strengthens the calls to support Indigenous food sovereignty initiatives, which entails First Nations regaining sufficient power to influence policies impacting traditional food systems, such as determining what should (and should not) happen in their land, watersheds, and territories [4, 7, 17], as a way to enhance well-being and cultural connectedness and potentially reduce health inequities in Canada [58].

## Data Availability

The datasets generated during the current study are not publicly available. Each community and the Indigenous Nation own their data. Any data request should be addressed to them through the corresponding author.
